# Agricultural climate change based on remote sensing image and emergency material supply management of agriculture, rural areas and farmers

**DOI:** 10.1007/s12517-021-07221-0

**Published:** 2021-05-17

**Authors:** Di Xu

**Affiliations:** School of Marxism, Jiangnan University, Wuxi, 214122 Jiangsu China

**Keywords:** Remote sensing image, Agroclimate, Agriculture, rural areas and farmers, Supply management

## Abstract

In the process of agricultural development, climate change in different regions will have different degrees of impact on the allocation and use of agricultural resources. In most cases, climate change will affect the use of agricultural production technology. The energy and material needed in the process of crop growth are also provided by climate change. People must pay attention to climate change if they want to carry out agricultural production. Through people’s long-term practice, it can be seen that the development of agriculture is greatly affected by climate. Once the climate change is unfavorable to the development of agriculture, the total grain reserve in China will be affected, and there will be some problems in the food supply of the whole country. In order to effectively reduce the negative impact of climate change, this paper analyzes the climate problems in the process of agricultural production by using geographic information technology, discusses the ability of agricultural resources to cope with sudden disasters, and puts forward reasonable resource allocation suggestions to improve the utilization efficiency of resources.

## Introduction

In the process of crop planting, agricultural climate resources are an important prerequisite to ensure the healthy growth of crops. Natural resources provide a lot of necessary energy and nutrients for the growth of crops. Abundant agricultural climate resources can also ensure that crops have a good growth environment. The development of agricultural production is related to the food and clothing of all the people in China. If climate change brings bad effects, then our daily diet will also be threatened. In the process of agricultural production, people usually have no way to change the structure of agricultural climate resources in the natural environment. We can only analyze the terrain and climate characteristics of the region, integrate and utilize the resources that are conducive to the growth of crops, and select the planting types of crops according to the specific terrain characteristics, so as to ensure the healthy growth of crops. Climate change is a normal phenomenon in nature, which cannot be changed by human beings through external forces. Once the climate changes, the agricultural ecological environment and the structure of means of production in agricultural production areas will also change to a certain extent. If we cannot effectively deal with this change, food security in China is likely to have problems. Through the analysis of a variety of agricultural production and climate change data, we can see that climate change has the characteristics of wide range and long-term impact. Agricultural production activities all over the world will be affected by climate change. Climate change can affect the agricultural development of many countries and regions. In recent years, the destruction of ecological environment caused by human production activities has led to many negative impacts on climate change. Some areas with fragile ecological environment have no ability to adjust the adverse effects of climate change, and agricultural production activities will be restricted. In the current development, the issue of climate change has developed into a global problem, and various countries and regions have begun to pay attention to the impact of climate change. In the process of global climate change, governments of various countries and regions use professional technology to analyze and evaluate the resources in agricultural production activities. Through specific analysis, feasible suggestions can be put forward for regional agricultural production activities. In the future development, the structure of agricultural climate resources in different regions is an important content related to agricultural development. Relevant departments should grasp the temporal and spatial distribution of agricultural climate resources, effectively guide agricultural production activities, and help different regions to achieve orderly agricultural production. In the process of analyzing agricultural climate resources, people can more intuitively know the use of agricultural climate resources and can effectively explore the agricultural production potential in some areas. On the basis of analyzing the allocation of resources, people can also screen the crop types in the region, optimize the crop structure in the region, and improve the efficiency of agricultural production.

In the process of social development, some sudden disasters or illness will have an impact on people’s daily life. Once an emergency occurs, people need to use the reserved resources to deal with disasters. If people do not have enough resources, then people’s future life will also be affected. In people’s daily life, some sudden disasters may seriously affect people’s material distribution and use. A lot of what people need in life are created by the development of agriculture. We need to fully consider the allocation of resources for agriculture, rural areas and farmers. When novel coronavirus started to cause pneumonia in China in late 2019, the outbreak of the epidemic caused many problems in the allocation and utilization of resources in our country. In the process of responding to the epidemic situation, the government and relevant departments have mobilized a lot of resources, but some resources are special needs of the epidemic situation, and there is no way to meet the needs of the changes of the epidemic situation in a short period of time. In the process of epidemic prevention and control, some prevention and control materials must be used, but some materials such as masks and protective clothing are very scarce. Even though the purchase price has reached a high level, the problem of material scarcity is still not solved. In the process of material management and distribution by the state and relevant governments, the management of disaster emergency materials is an important content to ensure people’s safety (Huang et al. 2016). The relevant departments should recognize the shortcomings in the management, strive to use reasonable management methods, improve the efficiency of material management, and reduce the damage to the people caused by disasters to the greatest extent. In the process of managing emergency materials for agriculture, rural areas and farmers, we should carefully analyze the impact of different disasters on social development, summarize the characteristics and frequency of disasters, effectively rectify the means and methods of material management, and design a more effective management plan. In order to improve people’s ability to deal with sudden disasters, this paper analyzes the climate problems in the process of agricultural production by using remote sensing technology and strives to improve the efficiency of agricultural production. In addition, the management of materials related to agriculture, rural areas and farmers is also an important part of people’s healthy life. This paper also analyzes the management of materials for agriculture, rural areas and farmers, through specific research to improve the ability of material management and reduce the negative impact of sudden disasters on people.

### The meaning of agriculture, rural areas and farmers

In the analysis of the definition of the material of agriculture, rural areas and farmers, researchers fully consider the relationship between the relevant concepts of public goods and the material of agriculture, rural areas and farmers. Through specific analysis, we can know that the material of agriculture, rural areas and farmers is mainly produced in rural areas, but this material is not the private goods owned by farmers, which can be used to meet the needs of people’s survival and development in rural areas; it can also be used to meet the development needs of social public groups (Erasmi et al. 2014). There are some differences between the material of agriculture, countryside and farmers, and other social products. This kind of material is not competitive and will not exclude other social products.

First, in a sense, the material of agriculture, rural areas and farmers is actually a kind of public material. Like other public goods, it includes the characteristics of public goods. Through the analysis of the characteristics of public goods, we can see that the material of agriculture, rural areas and farmers is not competitive, which shows that people do not need to choose the materials of agriculture, rural areas and farmers through competition. The materials in the market are shared by people. As long as people are willing to choose the materials, they can be purchased. The market rules will not affect the existence of the materials. However, the personal belongings of rural people are different from those of agriculture, rural areas and farmers. If one person consumes something, another person will have no choice. In addition, the materials of agriculture, rural areas and farmers, like public goods, will not exclude other types of goods, because these two kinds of goods do not require people to pay actual money, and people do not need to consider the issue of price, so they will not have an impact on people's choice of other types of consumer products (Hutchinson et al. 2009). However, farmers' own private goods need to be purchased by farmers themselves with a certain amount of money, it will be mutually exclusive with other types of objects.

Second, there are differences between the marginal utility of agriculture, rural areas and farmers and the marginal cost of their own. In order to calculate the marginal cost of agriculture, rural areas and farmers, we only need to add up the marginal benefits of the three rural materials. In order to calculate the marginal cost of farmers' private goods, we need to consider the marginal income of each unit. In a sense, the marginal cost of farmers' private goods is equal to that of each unit (Richardson et al. 2013).

Third, although there are some differences between the private goods owned by farmers and the materials of agriculture, rural areas and farmers in essence, people need to rely on the materials of agriculture, rural areas and farmers to consume private goods, that is to say, the private goods in rural areas are provided by the development of the material of agriculture, rural areas and farmers. In rural areas, people's production and management are small-scale Sun and Qin (2016). The construction of some water conservancy facilities, the construction and use of transportation, the popularization of science and technology, and the development of education have regional characteristics, which leads to the strong dependence of people in rural areas on agriculture, rural areas and farmers. In most cases, the more prosperous the economic development in rural areas, the stronger the dependence of people on agriculture, rural areas and farmers.

### Meaning of emergency materials

In people's daily life, sudden disasters may lead to chaos of social development. In order to reduce the damage of disasters to people, people need to prepare enough materials to deal with disasters. The materials that people use to deal with sudden disasters can be collectively referred to as emergency materials (Rani et al. 2018). This type of materials is mainly used to solve some sudden natural disasters or a wide range of public health problems, to help injured people and protect people's daily life. In the process of social development, people will encounter a variety of types of disasters, so people need to prepare emergency supplies to ensure that people can timely mobilize materials in the process of disaster prevention and control, disaster response, recovery and damage (Ibrahim et al. 2015). When a disaster occurs, the reserve of emergency materials determines people's ability to deal with disasters and solve problems to a certain extent. If emergency materials can be deployed to the disaster site in the shortest time, then the damage of disaster people can be reduced to the greatest extent.

### Analysis on characteristics of emergency materials

In people's daily life, once unexpected disasters occur, people need to deploy emergency materials as soon as possible in order to ensure people's life safety and normal needs. When a sudden disaster occurs, the speed of emergency materials deployment determines the efficiency of rescue and relief. Many types of disaster treatment need to be carried out in a short time, so emergency materials must be ready at any time. Some serious disasters, such as forest fires, floods and other disasters, will also threaten people's lives and health. The emergency materials needed at the rescue site are also diverse (Estel et al. 2015). People should fully analyze the characteristics of different disasters and prepare enough emergency materials to ensure that the rescue work of disaster relief personnel can be carried out smoothly. Some people who are injured in the shelter need to be prepared for emergency treatment when the disaster happens. In a word, the types of emergency materials should be constantly supplemented according to the changes of disasters, and the efficiency of material allocation should be improved as much as possible in the actual rescue work.

Although it is necessary for people to prepare enough emergency materials in advance to deal with sudden events, we cannot accurately judge the specific time, the region, the impact scale and the actual impact on people, so it is difficult to timely deploy emergency materials. Some emergencies may also cause damage to road traffic, which may seriously affect the transportation efficiency of emergency materials. In addition, once the natural disaster occurs, there is no way to predict the amount of natural disasters, so there may be no way to predict the amount of natural disasters.

In the current development process, people have accumulated a lot of experience in disaster prevention and treatment, so when storing emergency materials, people will reserve more in a planned way, which can improve people's ability and efficiency in dealing with emergencies to a certain extent. Although early storage of emergency materials can improve the efficiency of disaster treatment, many emergency materials have a shelf life. If they are not used in time within the effective time, it may cause waste (Peng et al. 2011). In view of the problems in the storage process of emergency materials, people need to check the service life of the materials stored in the warehouse on time, and timely replace some emergency materials that have been used beyond the service life or have deteriorated, so as to ensure that effective emergency materials can be deployed in case of disasters, so as to prevent the hazards caused to people by the expiration and deterioration of emergency materials.

### Basic ideas and objectives for the development of Agrometeorology

#### (1) Guiding ideology of scientific development

In the future development, we need to take the development of modern agriculture as the main goal of agricultural production. In order to improve the speed and quality of agricultural development, we should make full use of meteorological knowledge to provide help for agricultural production activities. In the development of agriculture, we should fully consider people's needs and promote the progress of agricultural production with demand. In agricultural production, we should use more new science and technology, adhere to the use of science and technology to improve the efficiency of agricultural production, use science and technology to promote the innovation of agricultural development, but also use science and technology to strengthen the monitoring and measurement of meteorological changes, and accurately predict the changes of the atmosphere. In the process of development, it is necessary to build a disaster prevention and control and alarm system, combine disaster prevention and control with the effective utilization of agricultural climate resources, so as to ensure food security in China and prevent problems endangering food security (Ichii et al. 2002).

#### (2) Basic principles of discipline development

In the process of agricultural development, the knowledge of meteorology is very important for agricultural production. In the use of meteorological knowledge, people need to follow certain principles, including the principle of developing agricultural production according to people's needs, the principle of serving the development of modern agriculture, the principle of promoting innovation with science and technology, the principle of developing agricultural production according to the characteristics of the region, and the principle of combining the development of knowledge of multiple disciplines.

#### (3) Main objectives of discipline development

According to the development plan, we plan to use the knowledge of meteorology to establish a service system for the development of modern agriculture in 2020.

In the process of agricultural production, the prediction of meteorological changes can help people to judge the appropriate production time and place to a great extent. When using meteorological knowledge and technology to guide agricultural production activities, it is necessary to determine the direction of agricultural development is the development direction of modernization, and make full use of science and technology to improve the efficiency of production (Patel et al. 2015). People can use advanced science and technology to build weather simulation model, which can more effectively analyze various problems in the process of agricultural production, and improve the modernization level of China's agricultural development.

## Materials and methods

### Data sources

#### Climate data

**Fig. 1.**
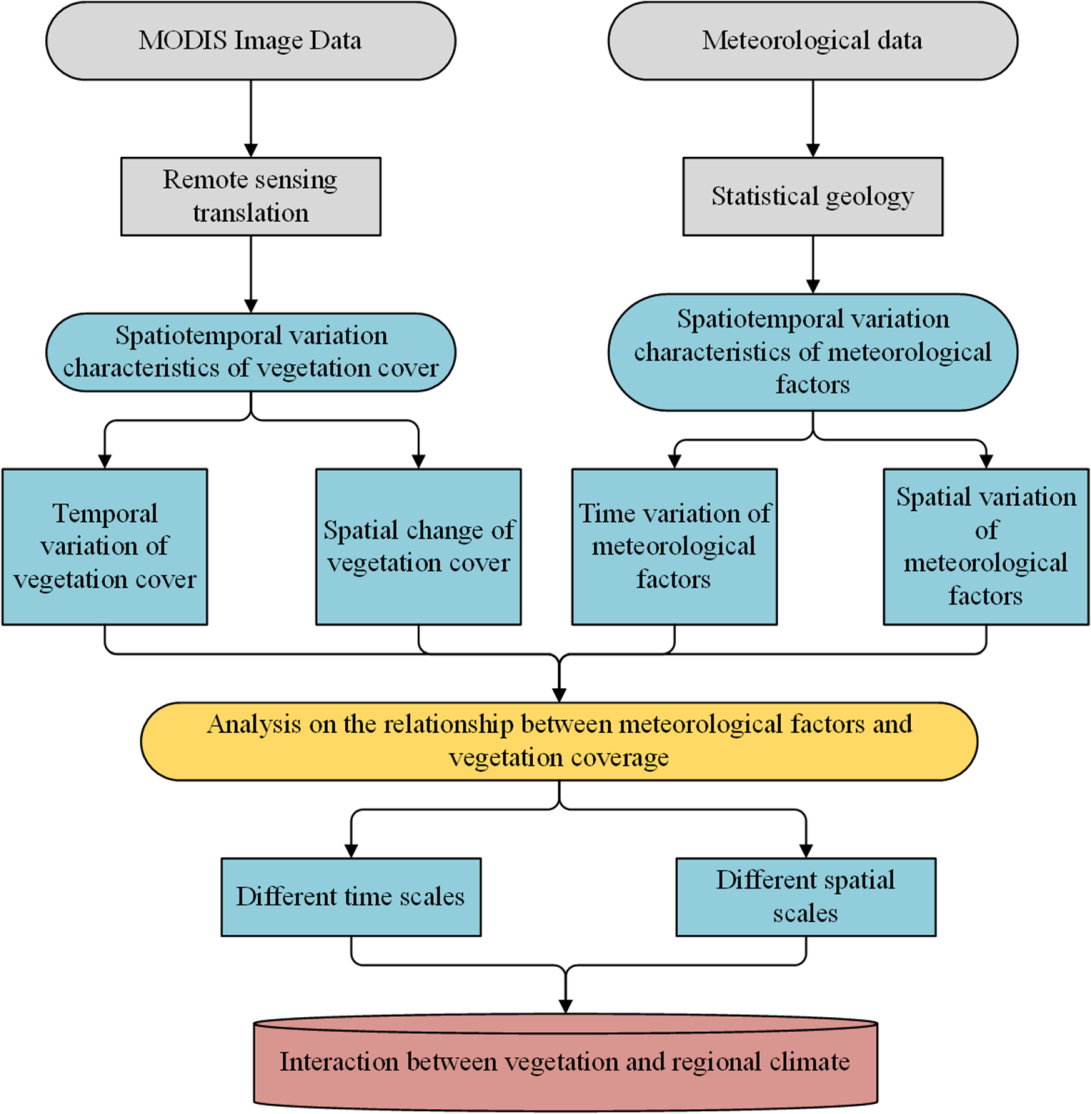
Data analysis technology roadmap

The data used in this study is mainly collected from China's Meteorological websites. The main purpose of collecting relevant information and data is to analyze the impact of climate warming on the growth of crops (Olthof et al. 2015). This study analyzes the data detected by the meteorological stations in a certain province, and processes the collected information and data with special software, as shown in Figure 1. It can meet the needs of research.

#### Remote sensing image data

In the process of agricultural development, the use of remote sensing technology can improve people's ability to prevent disasters. This paper collected remote sensing images collected by multiple remote sensing sensors to analyze the growth of crops. The collected images were processed by professional software, and the key data needed for research were extracted (Feng et al. 2016a).

#### Other data statistics

To specifically analyze the situation of agricultural production, we also need to collect the data of crop planting in a certain area, and count the types and planting areas of different crops. By analyzing the planting situation of crops, we can know the spatial distribution of crops directly (Kendall 1975). In this study, the area of crops sown in a certain area was counted, and the size of planting area was used as an index to evaluate the economic benefits of crops. Through further analysis, the relationship between the growth of crops and climate change can be obtained.

### Data processing

#### Processing methods of remote sensing data

In the process of analysis, we need to use professional software to process the collected remote sensing images and extract the data needed by the research Cai and Yu (2009). By processing, people can calculate the gray value of remote sensing data. In order to meet the needs of research, people also need to analyze the product description, convert the gray value, and use the maximum value synthesis method to process the gray value. The data set of NDVI can be obtained. The specific calculation formula is as follows:

1 $$ {\Theta}_{allope}=\frac{n\sum \limits_{i=1}^n{iX}_t-\left(\sum \limits_{i=1}^ni\right)\left(\sum \limits_{i=1}^n{X}_t\right)}{n\sum \limits_{i=1}^n{i}^2-{\left(\sum \limits_{i=1}^ni\right)}^2} $$

#### Sorting and processing methods of meteorological data

Because agricultural production activities cannot do without meteorological information monitored by meteorological stations, the analysis of agricultural development needs to analyze meteorological data. The collected data are used to calculate the impact of different meteorological factors on agricultural production activities by using the method of monthly meteorological data set (Kim et al. 2014; Ning et al. 2018).

(1) Linear tilt rate is an important meteorological factor. In the calculation, we need to pay attention to the influence of time change on the numerical value (Guan et al. 2019). The calculation of this factor can be carried out directly by using Excel.

(2) Sliding smoothing rate is also an important part of meteorological data analysis. To calculate its size, the data needs to be smoothed, and the growth of crops can be judged by analyzing the change of value.

(3) In this paper, the method of sorting is used to analyze climate change:

2 $$ {S}_k=\sum \limits_{i=1}^k{r}_i\left(k=2,3,\dots, n\right) among,{r}_i=\left\{\begin{array}{l}+1\  when\ {x}_i>{x}_j\left(j=1,2,\dots, i\right)\\ {}0\  otherwise\end{array}\right\} $$

Next, define the size of the quantity:

3 $$ {UF}_k=\frac{S_k-E\left({S}_k\right)}{\sqrt{Var\left({S}_k\right)}}\left(K=1,2,\dots n\right) $$

(4) The spatial interpolation is used to analyze the spatial variation of meteorological factors.

(5) The use of linear regression equation can effectively analyze the meteorological changes, through the analysis of the change of meteorological factors; we can see the situation of vegetation coverage (Feng et al. 2016b).

(6) The calculation of the linear correlation coefficient can help people to analyze the correlation between meteorological factors, and the formula is as follows:

4 $$ r=\frac{\sum \limits_{i=1}^n\left({x}_i-\overline{x}\right)\left({y}_i-\overline{y}\right)}{\sqrt{\sum \limits_{i=1}^n{\left({x}_i-\overline{x}\right)}^2\cdot \sum \limits_{i=1}^n{\left({y}_i-\overline{x}\right)}^2}} $$

### Methods of climate analysis in agricultural season

The linear regression equation established in this study is as follows:

5 $$ {\hat{x}}_i=a+{bt}_i\left(i=1,2,\dots, n\right) $$

6 $$ b-\frac{\sum \limits_{i=1}^n\left({t}_j-\overline{t}\right)\left({x}_i-\overline{x}\right)}{\sum \limits_{i=1}^n{\left({t}_i-\overline{t}\right)}^2} $$

During the analysis, the inverse distance weight interpolation method can be used to analyze the data monitored by different weather stations Milich and Weiss (2000). The calculation formula is as follows:

7 $$ r-\frac{\sum \limits_{i=1}^n\left({t}_j-\overline{t}\right)\left({x}_i-\overline{x}\right)}{\sum \limits_{i=1}^n{\left({t}_i-\overline{t}\right)}^2\sum \limits_{i=1}^n{\left({x}_i-\overline{x}\right)}^2} $$

## Results

### Spatial distribution characteristics of agricultural vegetation cover based on remote sensing images

**Fig. 2:**
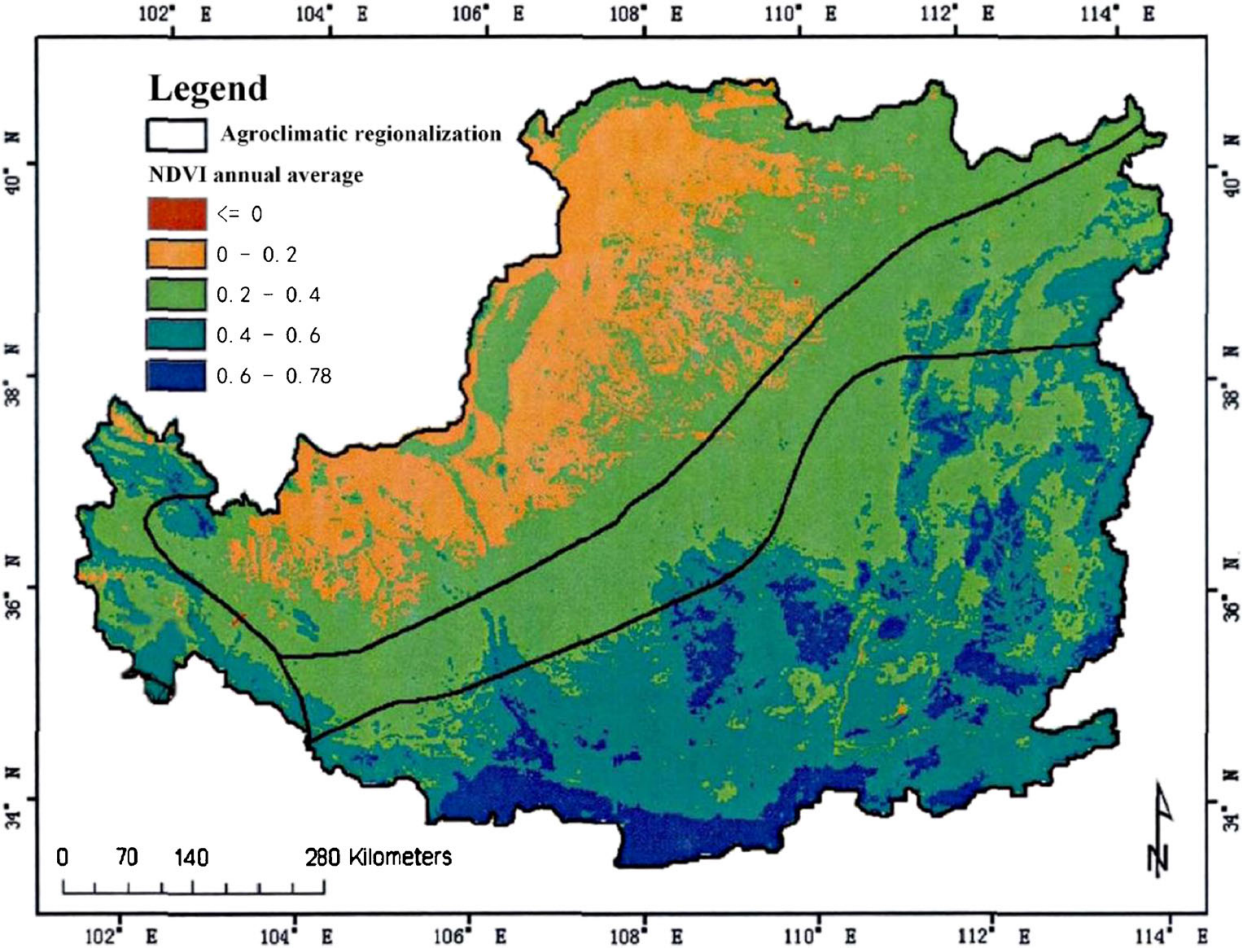
Spatial distribution of NDVI in 2000–2019 in a plateau area

By observing Fig. 2, we can see the distribution of NDVI values in a certain plateau area from 2000 to 2019.

**Fig. 3:**
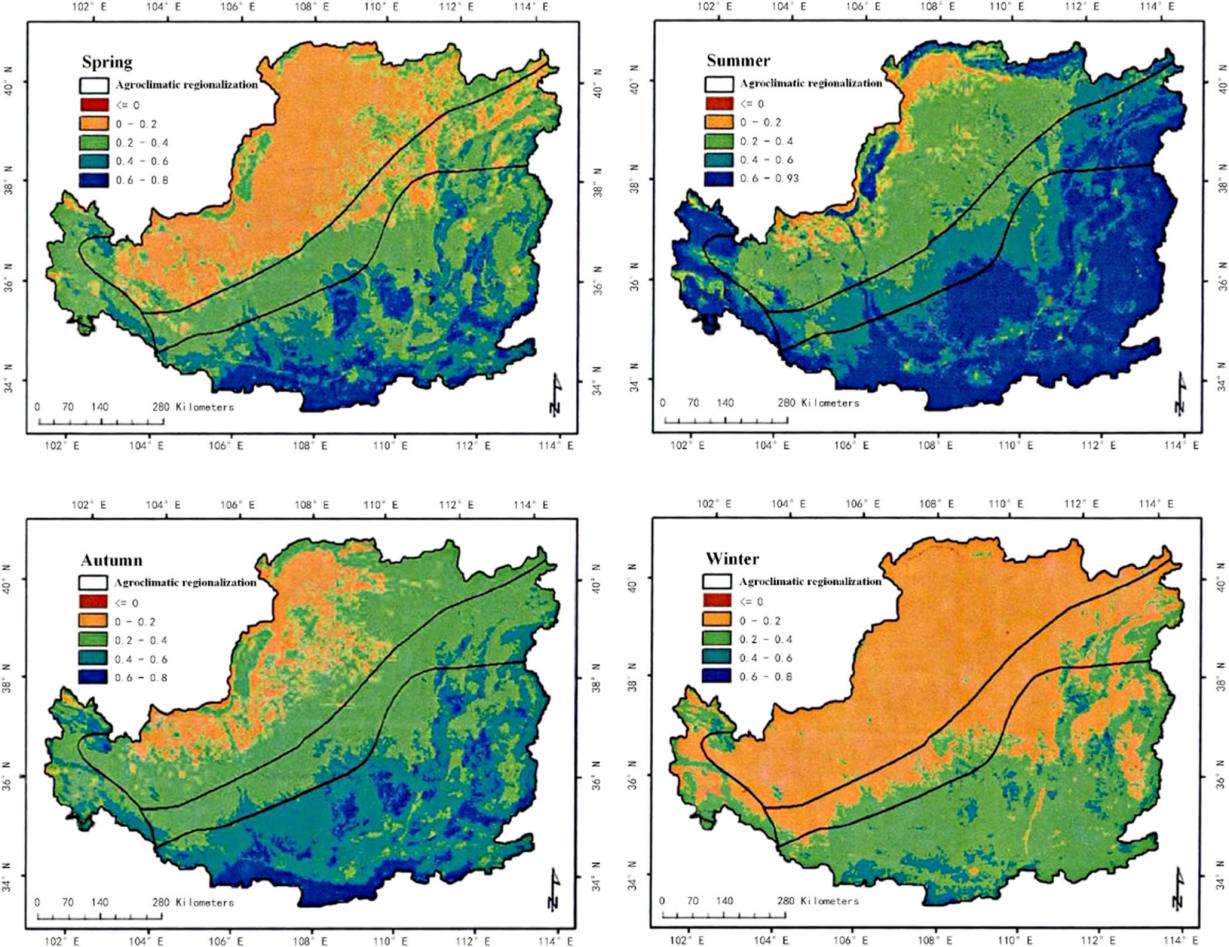
The spatial distribution of NDVI mean values in spring, summer, autumn, season, and winter in a plateau area from 2000 to 2019

**Fig. 4:**
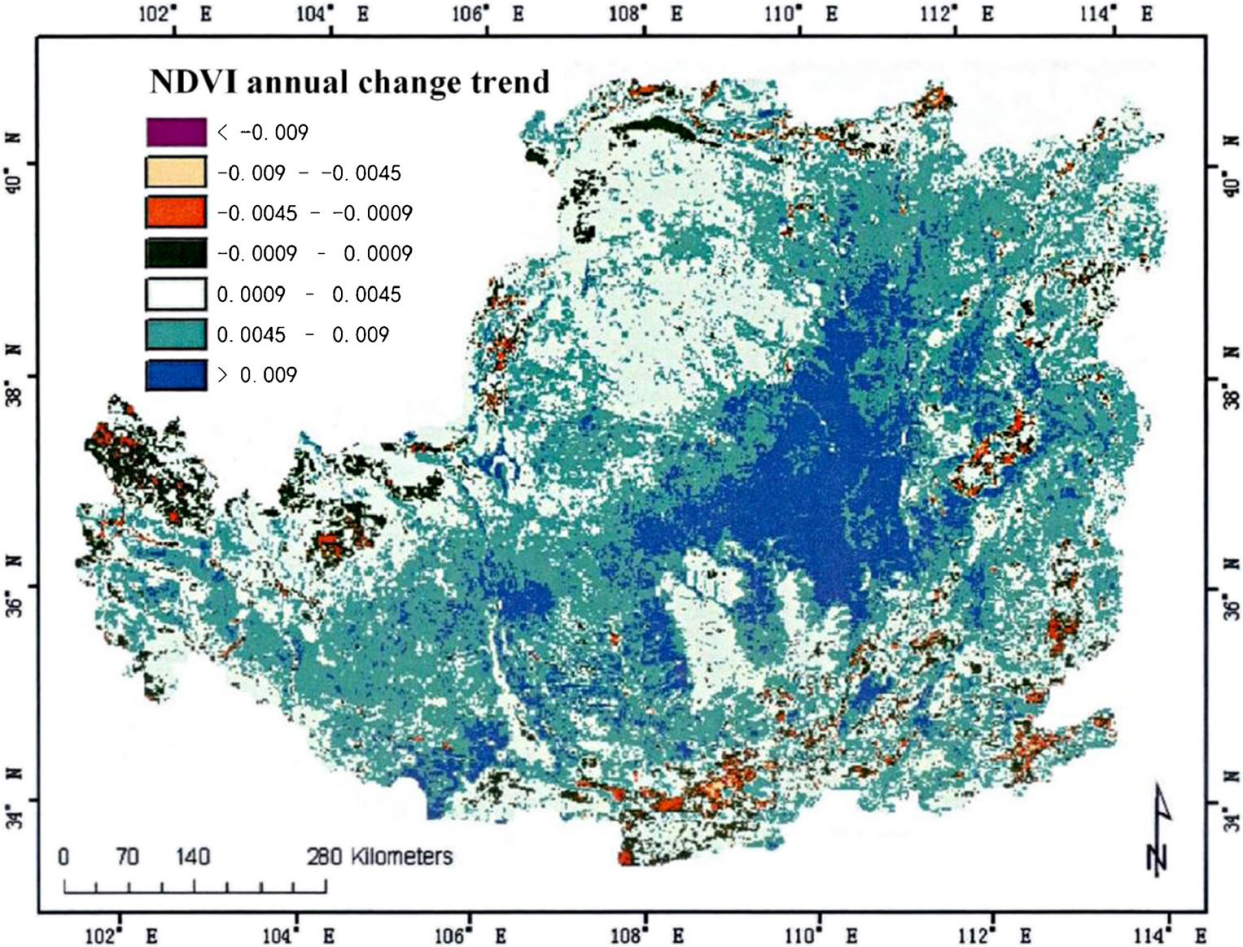
Annual NDVI change trend of a plateau from 2000 to 2019

**Fig. 5:**
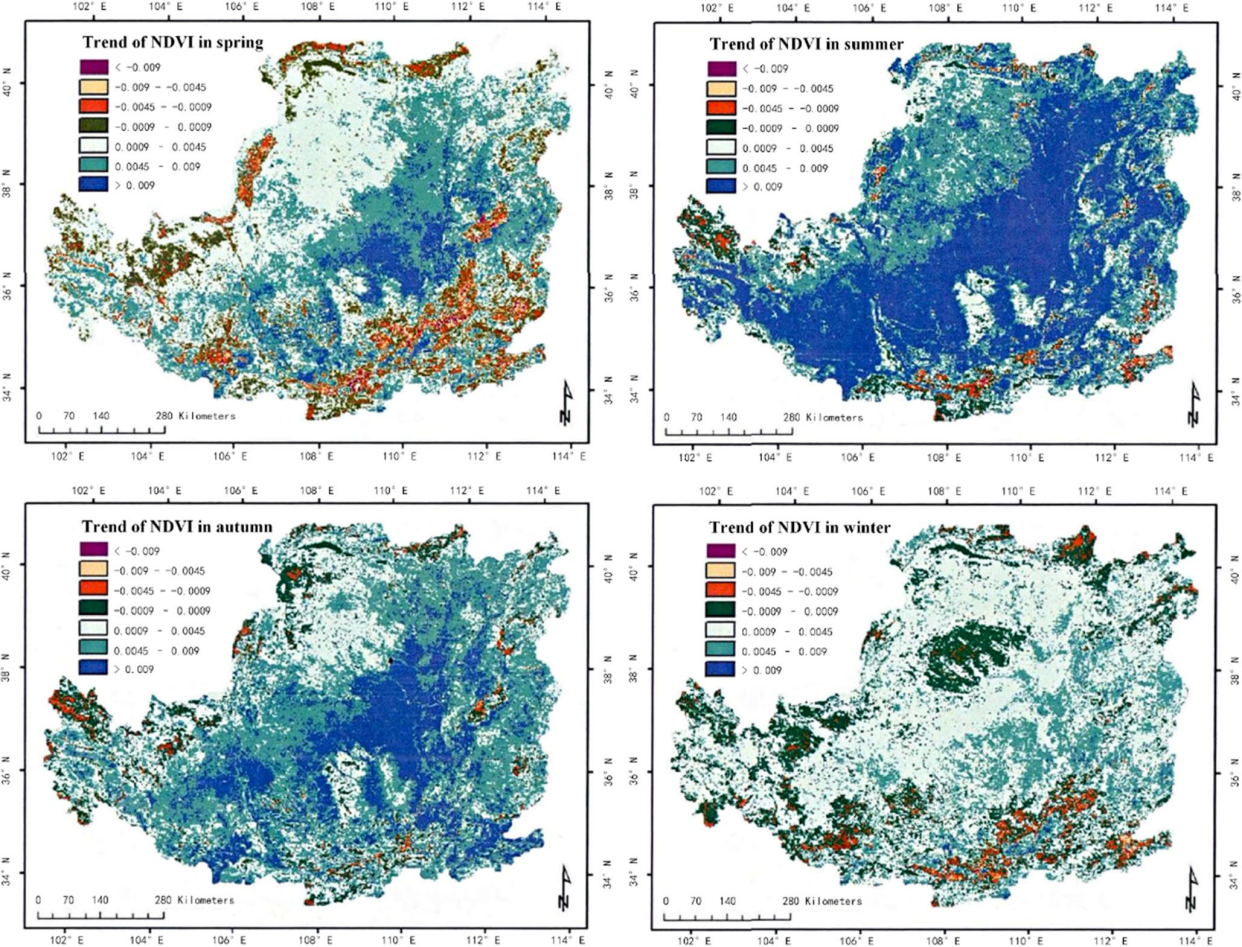
Variation trend of NDVI in different seasons of a plateau in recent 19 years

In the analysis of climate change, we can analyze the development of the region by observing the vegetation coverage in different areas (Lee et al. 2016). This paper calculates the vegetation coverage of a certain plateau in different seasons. The specific situation is shown in Fig. 3, Fig. 4 and Fig. 5.

In order to analyze the vegetation coverage, the NDVI values in different months were analyzed. The specific results are shown in Fig. 3.

**Fig. 6:**
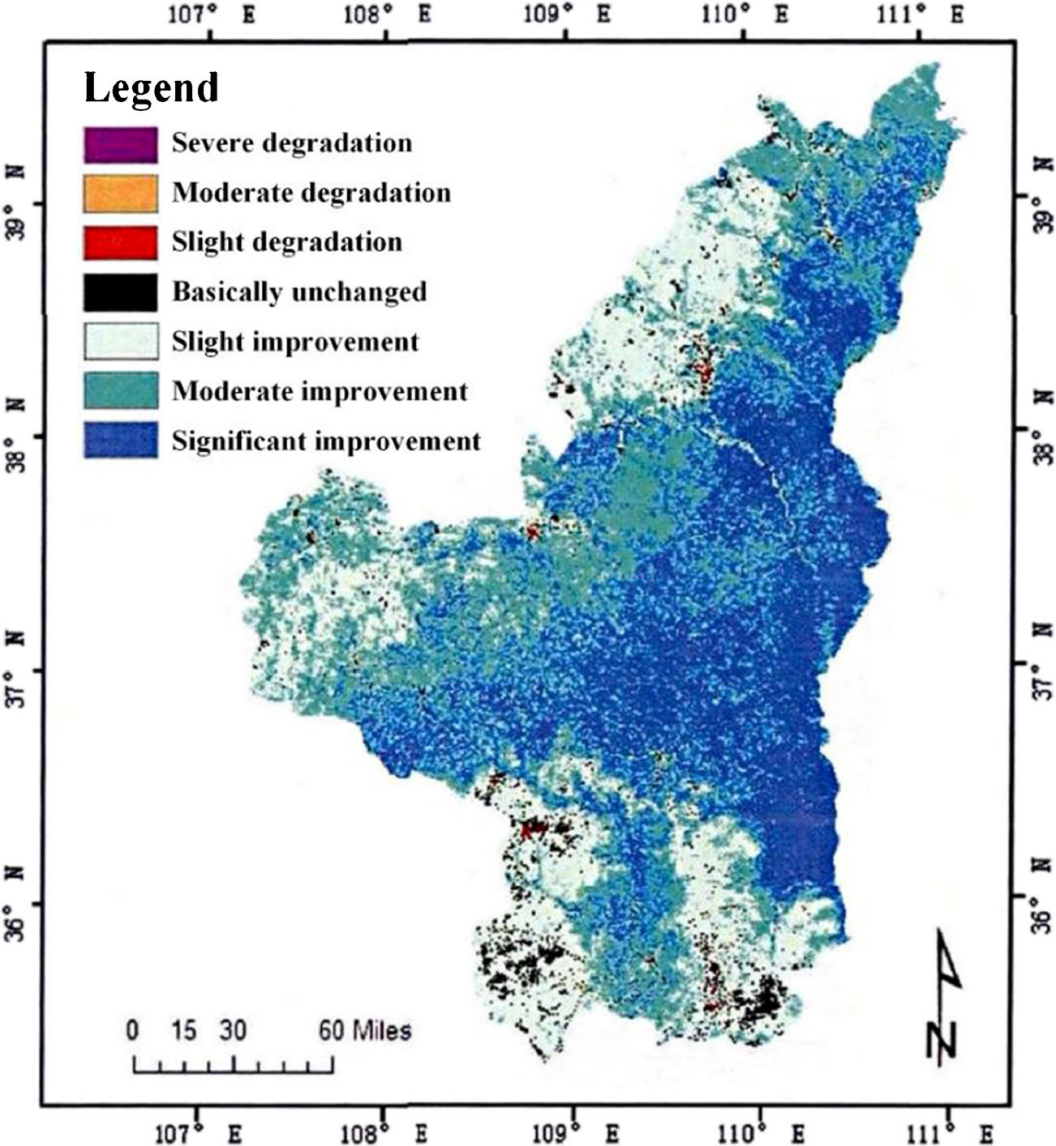
Spatial distribution map of annual NDVI change trend in Northern Shaanxi of a plateau

**Fig. 7:**
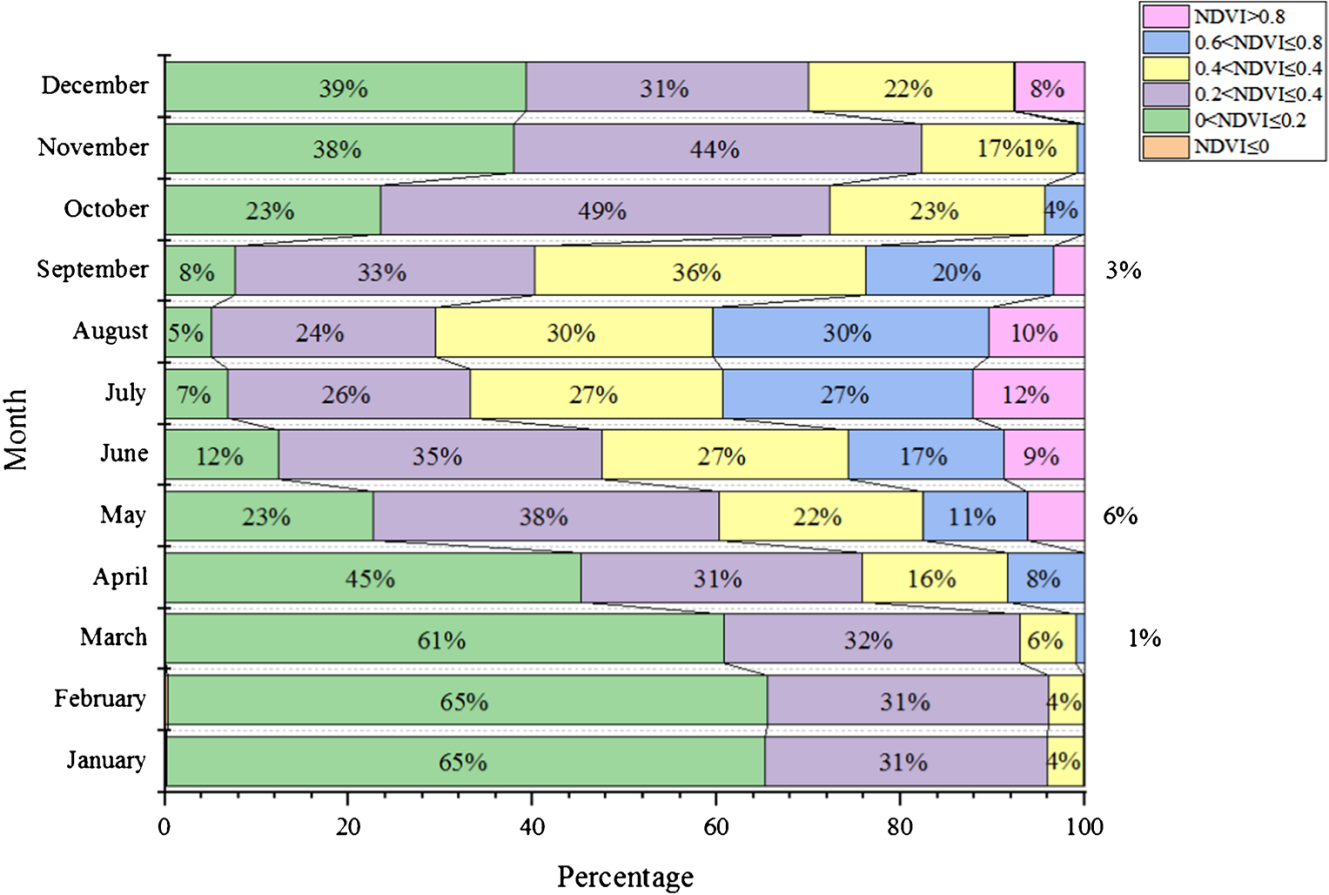
Statistics of each level of the average monthly NDVI in a plateau area from 2000 to 2019

**Fig. 8:**
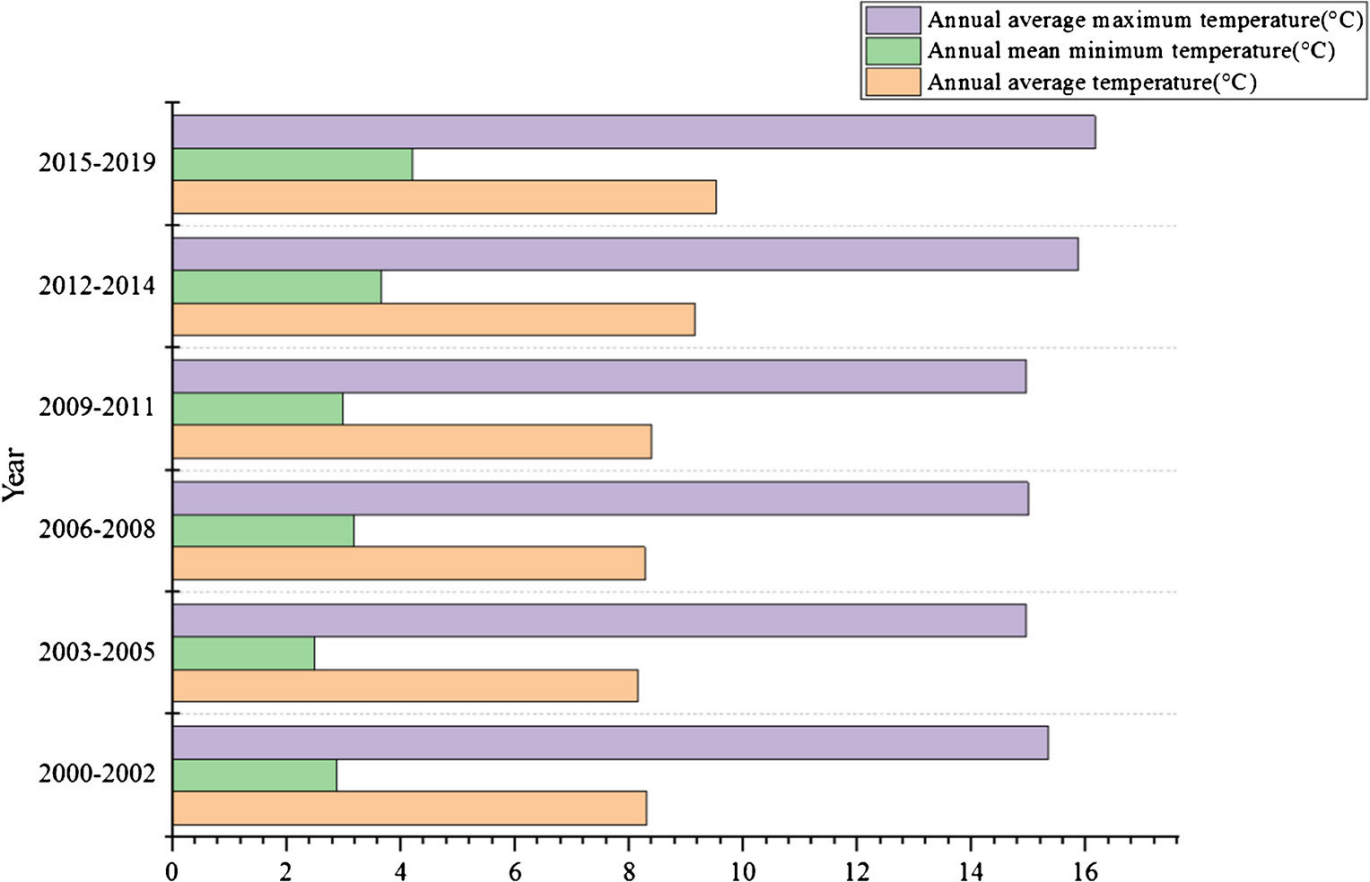
Interdecadal variation of annual average temperature, minimum temperature, and maximum temperature in a plateau area

**Fig. 9:**
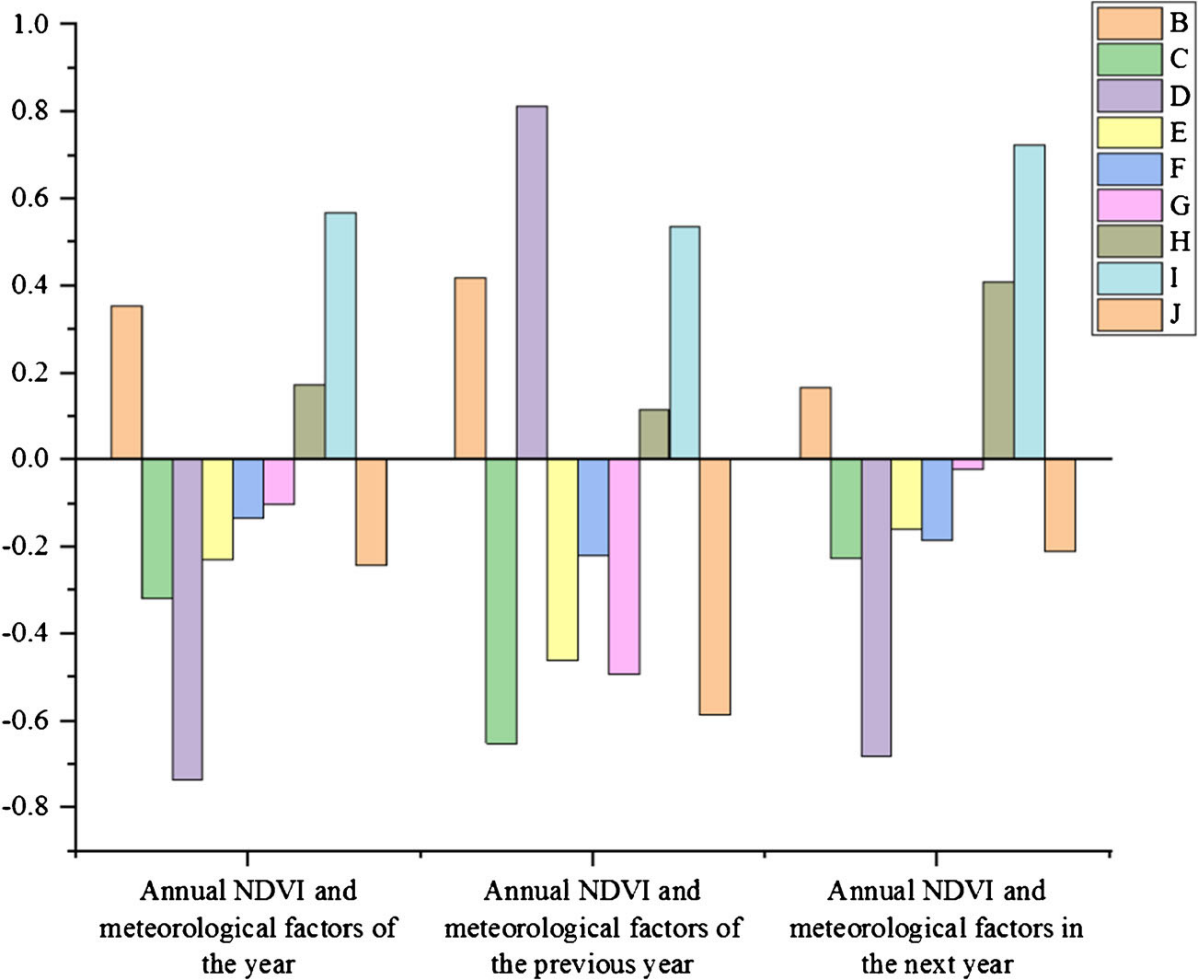
Correlation analysis results of annual average NDVI and annual meteorological factors in a plateau from 2000 to 2019

**Fig. 10:**
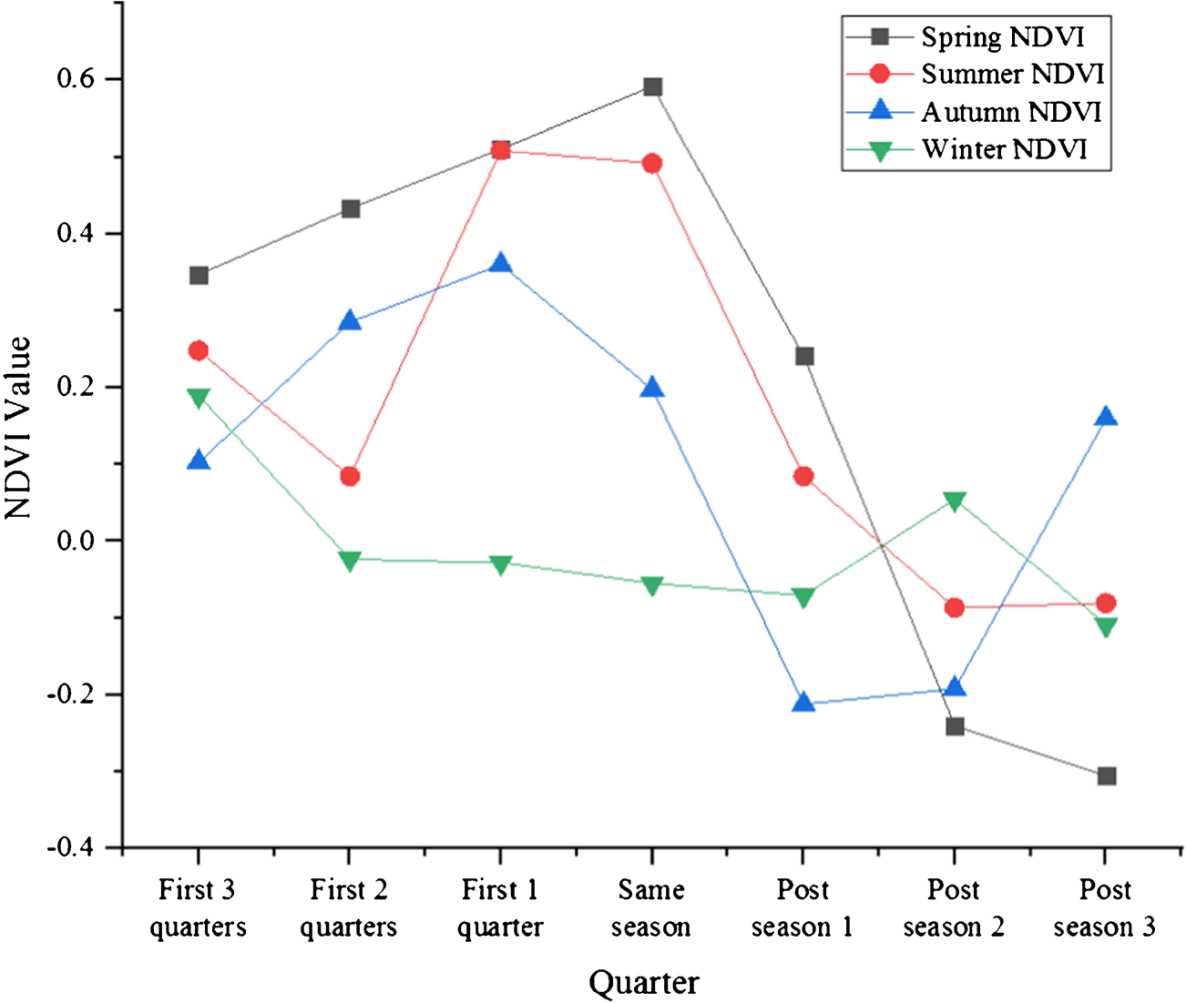
Correlation analysis results of seasonal average NDVI and seasonal precipitation in a plateau from 2000 to 2019

This study also analyzed the vegetation distribution of a certain plateau in different months, and the specific situation is shown in Fig. 6, Fig. 7, Fig. 8, Fig. 9, and Fig. 10. In the process of analysis, NDVI values of different months are also calculated, and the results are shown in Table 1.

**Table 1:** Statistics of NDVI mean values of different grades in a plateau area from 2000 to 2019

Month	Area percentage%					
January	NDV≤0	0<NDVI≤0.2	0.2<NDVI≤0.4	0.4<NDVI≤0.4	0.6<NDVI≤0.8	NDVI>0.8
February	0.20	65.09	30.62	3.93	0.16	
March	0.29	65.27	30.57	3.82	0.05	
April	0.12	60.63	32.26	6.01	0.99	
May	0.07	45.13	30.48	15.82	8.39	
June	0.04	22.67	37.55	22.11	11.43	6.20
July	0.03	12.34	35.16	26.73	16.97	8.77
August	0.02	6.84	26.37	27.38	27.24	12.14
September	0.02	5.07	24.28	30.22	29.99	10.42
October	0.03	7.56	32.65	35.96	20.37	3.42
November	0.1J	23.42	48.82	23.40	4.26	
December	0.11	37.86	44.35	16.91	0.77	

### Variation trends of meteorological factors over time based on remote sensing images

The change data of precipitation is shown in Table 2.

**Table 2:** Interdecadal variation of precipitation in a plateau area (unit: mm)

E	2000–2002	2003–2005	2006–2008	2009–2011	2012–2014	2015–2019
Average wind speed	2.21	2.44	2.53	2.20	2.22	2.09
Average maximum wind speed	12.64	14.74	12.31	11.34	10.45	9.69

Observing the data in Table 3, we can know that the precipitation in a plateau area has been decreasing during the long-term development, and overall, there is a clear downward trend.

**Table 3:** Interdecadal variation of annual average wind speed and maximum wind speed in a plateau area (unit: m/s)

Years	2000–2002	2003–2005	2006–2008	2009–2011	2012–2014	2015–2019
Precipitation	504.90	505.41	476.44	487.84	453.11	479.44

When analyzing meteorological data, this study also analyzed the changes in average wind speed and maximum wind speed in different years in the plateau area (Leroux et al. 2017). The specific data are shown in Table 4.

**Table 4:** Interdecadal variation of annual average temperature, minimum temperature and maximum temperature in a plateau area (unit: °C)

Years	2000–2002	2003–2005	2006–2008	2009–2011	2012–2014	2015–2019
The annual average temperature	8.31	8.16	8.28	8.39	9.16	9.53
Annual average minimum temperature	2.88	2.49	3.18	2.98	3.65	4.21
Annual average maximum temperature	15.35	14.96	14.99	14.96	15.86	16.16

After analyzing the changes in precipitation and wind speed, this study also conducted statistics and analysis on temperature changes in a plateau area. The statistical results are shown in Table 5.

**Table 5:** Interdecadal changes of annual average relative humidity and average minimum relative humidity in a plateau area (unit: %)

Years	2000–2002	2003–2005	2006–2008	2009–2011	2012–2014	2015–2019
Average relative humidity	62.59	59.89	59.14	59.45	58.80	57.54
Average minimum relative humidity	17.30	16.34	16.69	13.71	13.91	13.64

By observing the data in Table 6, we can know that the relative humidity of a plateau area has been decreasing for a long time, and the water in this area has been losing.

**Table 6:** Interdecadal variation of sunshine hours in a plateau area (unit: hour)

Years	2000–2002	2003–2005	2006–2008	2009–2011	2012–2014	2015–2019
Sunshine hours	2335.24	2612.36	2606.80	2482.98	2499.75	2445.60

### The relationship between agricultural vegetation coverage and meteorological factors based on remote sensing images

#### The relationship between annual average NDVI and meteorological factors

By analyzing the changes of meteorological factors, the changes of the annual average NDVI value in a plateau area can be explained. The specific conditions are shown in Table 7.

**Table 7:** Correlation coefficients between average annual NDVI and annual meteorological factors in a plateau from 2000 to 2019

Related items	Annual precipitation	Average annual wind speed	Maximum annual wind speed	Average annual temperature	Annual minimum temperature	Annual Maximum temperature	Annual average relative humidity	Annual minimum relative humidity	Average annual sunshine hours
Annual average NDVI and meteorological factors of the year	0.356	− 0.318	− 0.736	− 0.230	− 0.135	− 0.100	0.173	0.568	− 0.241
Average annual NDVI and meteorological factors in the previous year	0.419	− 0.652	0.814	− 0.461	− 0.218	− 0.494	0.115	0.536	− 0.587
Annual average NDVI and meteorological factors in the next year	0.167	− 0.225	− 0.681	− 0.159	− 0.185	− 0.021	0.408	0.725	− 0.209

#### The relationship between vegetation NDVI and meteorological factors

In the development process of a plateau area, the change of precipitation will also have a certain impact on the NDVI value of the plateau area (Li et al. 2016). The specific analysis is shown in Table 8.

**Table 8:** Correlation coefficient between seasonal average NDVI and seasonal precipitation in a plateau from 2000 to 2019

Seasonal NDVI	Precipitation						
	First 3 quarters	First 2 quarters	First 1 quarter	Same season	Post season	Post season 2	Post season 3
Spring NDVI	0.347	0.433	0511	0.592	0.241	− 0.240	− 0.305
Summer NDVI	0.248	0.085	0.509	0.492	0.085	− 0.087	− 0.081
Fall NDVI	0.103	0.285	0.360	0.198	− 0.212	− 0.192	0.160
Winter NDVI	0.19	− 0.023	− 0.028	− 0.055	− 0.071	0.055	− 0.109

Observing the data in Table 9 can see the changes in NDVI values in different seasons in a plateau area.

**Table 9:** The correlation coefficient between the seasonal average NDVI and the seasonal average wind speed of a plateau from 2000 to 2019

Seasonal NDVI	First 3 quarters	First 2 quarters	First 1 quarter	Same season	Post season	Post season 2	Post season 3
	Average wind speed						
Spring NDVI	− 0.196	− 0.477	− 0.424	− 0.705	− 0.442	− 0.077	-0.082
Summer NDVI	− 0.371	− 0.265	− 0.589	− 0.383	− 0.019	− 0.092	-0.352
Fall NDVI	− 0.129	− 0.447	− 0.255	− 0.236	-0.070	− 0.516	-0.190
Winter NDVI	-0.008	-0.190	0.033	0.381	0.000	− 0.09	-0.346
Maximum wind speed							
Spring NDVI	− 0.121	− 0.412	− 0.573	− 0.807	− 0.482	− 0.083	− 0.434
Summer NDVI	9.424	− 0.667	− 0.642	− 0.596	− 0.613	− 0.593	− 0.520
Fall NDVI	− 0.462	− 0.530	− 0.638	− 0.490	− 0.548	− 0.633	− 0.237
Winter NDVI	− 0.074	− 0.291	− 0.002	− 0.152	− 0.184	− 0.209	− 0.46

The influence of temperature on the NDVI value of a plateau area is shown in Table 10.

**Table 10:** Correlation coefficient between seasonal average NDVI and seasonal average temperature of a plateau from 2000 to 2019

Seasonal NDVI	First 3 quarters	First 2 quarters	First 1 quarter	Same season	Post season	Post season 2	Post season 3
Average temperature							
Spring NDVI	− 0301	− 0.069	0254	0.169	0.284	− 0.079	− 0.072
Summer NDVI	− 0.015	− 0.309	0.068	− 0.035	0.023	− 0.077	0.244
Fall NDVI	− 0.124	− 0.201	0.136	0.197	0.272	0.182	− 0.124
Winter NDVI	− 0.195	− 0.004	0.281	0.511	0.057	0.195	− 0.182
Maximum temperature							
Spring NDVI	− 0.223	− 0.123	0391	0.014	− 0.304	0.318	− 0.001
Summer NDVI	− 0.004	0.375	− 0.029	− 0.179	− 0.273	0.038	0.269
Fall NDVI	− 0.027	− 0.175	0.266	0.212	− 0.151	0.235	− 0.193
Winter NDVI	− 0.084	− 0.026	0.245	0.491	0.085	0.112	− 0.074
Minimum temperature							
Spring NDVI	− 0.246	0.061	0.068	0.478	− 0.103	− 0.297	− 0.089
Summer NDVI	0.061	− 0.159	0.306	0.280	− 0.111	− 0.130	0.166
Fall NDVI	− 0.029	− 0.100	0.159	0.214	-0.316	0.035	0.073
Winter NDVI	− 0.229	0.042	0.242	0.486	-0.063	0.284	− 0.147

This study also analyzed the relationship between vegetation coverage index and temperature in different seasons. The results of the analysis are shown in Table 11.

**Table 11:** Correlation coefficient between seasonal average NDVI and seasonal average relative humidity in a plateau from 2000 to 2019

Seasonal NDVI	First 3 quarters	First 2 quarters	First 1 quarter	Same season	Post season	Post season 2	Post season 3
Average relative humidity							
Spring NDVI	− 0.211	0.193	0.579	0307	0.027	0.514	− 0.422
Summer NDVI	− 0.041	0.170	0.110	0.169	0.481	0.579	− 0.308
Fall NDVI	0.115	0.016	0.152	0.008	0.432	0.376	− 0.024
Winter NDVI	0.028	0.125	0.096	0.032	0.418	− 0.094	− 0.114
Minimum relative humidity							
Spring NDVI	− 0.410	− 0.272	0.217	0.146	0.205	0.191	0.121
Summer NDVI	− 0.274	0.182	0.278	0.071	0.622	− 0.406	− 0.178
Fall NDVI	− 0.485	− 0.384	0.018	0.412	0.573	0.658	− 0.284
Winter NDVI	− 0.144	0.060	− 0.007	0.142	0.514	− 0.402	− 0.378

## Discussion

### Temporal and spatial changes of agricultural vegetation cover based on remote sensing images

Using the vegetation index data for the past 19 years, discuss the surface vegetation coverage of a plateau terrain area, which mainly includes the characteristics of vegetation coverage in different locations and the changes in time and space. The results of the discussion are as follows:

(1) In the past 19 years, the vegetation coverage of this plateau terrain has changed significantly in space. The vegetation index can see distinct stratification. This stratification shows a trend of gradual decrease from the southeast to the northwest. It is divided into three levels, which are consistent with the changes in my country’s temperature zones. This also shows that the plateau area is vast (Duarte et al. 2015). The vegetation in this terrain area changes due to the four seasons, and the vegetation coverage in each season will also change significantly. The coverage is best in summer, followed by spring and autumn, and the worst in winter; of course, we can also observe the changes in season vegetation coverage of each month, the vegetation coverage is good in the hot months, and the cold months are poor (Fu et al. 2017). The vegetation coverage in different months is also consistent with the stratification trend in spatial changes, and it is also gradually from southeast to northwest. Decrease, which can also be seen in July and August when the weather with the best vegetation coverage is hotter, the best indicator of the vegetation index in this terrain area.

(2) In the past 19 years, the vegetation coverage of this plateau terrain area has also changed significantly over time. The overall vegetation coverage of this area has improved to a better trend, but this trend is only in the spring and winter when the weather conditions are more severe. Shows a relatively small improvement, and this trend is particularly significant in summer when weather conditions are good and high temperatures, and this trend change is also relatively significant in autumn when weather conditions are better. According to the characteristics of seasonal changes, it can also be seen that this trend change is the most obvious in July when the weather is hot, and the opposite is the slightest in December.

(3) In the past 19 years, the vegetation coverage of this plateau terrain area has shown a certain degree of difference and hierarchy in space. The overall vegetation coverage of this terrain area has increased, but this trend is in poorly improved areas (Wang et al. 2015). Areas with better improved conditions are followed. The most obvious area with increased vegetation coverage is the middle of the plateau terrain. However, areas with poorer and better improved conditions account for the largest area of this terrain. The remote areas of the terrain area and parts of the southeast are areas with little improvement in vegetation coverage.

### The impact of meteorological factors on agricultural vegetation coverage

According to the climate change of the terrain area, we can observe the temporal and spatial characteristics of the plateau terrain area under different weather conditions

1. In recent years, the precipitation in the plateau terrain area has shown a downward trend, but the downward trend is not very obvious. In recent 19 years, the plateau terrain area has a relatively obvious upward trend, and most of the terrain area has shown this upward trend.
2. In recent years, the fractional change of the plateau terrain area is also very obvious, or a downward trend, in which the average wind speed is less than the maximum wind speed. In recent years, both the average wind speed and the maximum wind speed have changed significantly. Therefore, in the last 19 years, the change of the terrain area has shown an obvious downward trend, whether it is the average wind speed or the maximum wind speed. It can also be seen from the space that this downward trend takes up a large proportion in the plateau terrain area.
3. In recent years, the temperature change of the plateau terrain area is also very significant, whether it is the highest temperature, the lowest temperature, or the average temperature, showing an obvious upward trend. In recent years, the temperature in the plateau terrain area has a most obvious change trend, in which the maximum temperature, minimum temperature, and average temperature in most areas show a downward trend, but the change trend of minimum temperature is the least significant (Wang et al. 2019). However, according to this change, it can also be seen that the areas with significantly improved vegetation coverage are more consistent with the areas with obvious downward trend of maximum temperature.
4. In recent years, the humidity of the plateau terrain area also showed a downward trend, in which the trend did not change significantly in the years, the change trend of humidity decline in recent 19 years is relatively gentle.
5. Through the relevant data, it can be seen that the illumination time of the plateau terrain area has obvious changes, showing a regional downward trend, but in recent years, there are obvious changes in years. In recent 19 years, the light duration of the plateau terrain area has shown an obvious downward trend, and this downward trend can be seen in most areas of the plateau terrain area, accounting for a large proportion. According to the changes of different climates above, it can be seen that the climate of the plateau terrain area is developing in the direction of dry and hot (Gandhi et al. 2015). Although the precipitation in the plateau terrain area has shown an increasing trend in the recent 19 years, the humidity still shows a downward trend and has not been significantly improved. However, in general, due to the implementation of the national policy of returning farmland to forest in the past 19 years, the government has implemented the policy of returning farmland to forest, and the climate of the plateau terrain area has been improved.

### Measures and countermeasures for the development of agrometeorology

#### Strengthen the application of new technologies in agrometeorology

Satellite remote sensing image technology plays an important role in the development of agriculture. It can timely report meteorological changes, early warning of meteorological disasters and comprehensive emergency measures, monitor the growth of crops, and plan areas with poor weather conditions. At the same time, we should strengthen the support of big data and network resources and improve the impact of agricultural meteorology and risk prevention. At the same time, it is necessary to enhance the application of satellite remote sensing monitoring system and image processing technology to monitor the growth of crops in an all-round and all-weather way. One of the most important is to make good use of satellite remote sensing technology in weather forecasting, multi-level and multi angle to promote the development of agriculture, to avoid the impact of meteorological disasters and other related meteorological changes.

#### Strengthen the foundation of automatic observation of agricultural meteorology

We should establish and improve an all-round, all-weather, multi angle, professional, and intelligent agricultural meteorological monitoring system, upgrade and strengthen the monitoring technology and equipment, adjust the location and distribution range of monitoring instruments, adjust and optimize the construction of monitoring system, and focus on strengthening the specialized and intelligent agricultural meteorological monitoring system. In order to better promote the development of agriculture, intelligent monitoring network should be constructed to update crop growth and local climate change in real time (Liu et al. 2018). At the same time, we should continue to strengthen the research and development and optimization of agricultural meteorological monitoring system and establish a monitoring system with the same terminal to forecast natural disasters and the natural environment of agricultural growth, so as to reduce the impact of meteorological disasters on crop growth.

#### Develop practical technology of modern agricultural meteorology

Nowadays, the adjustment of agricultural industrial structure is pursuing more characteristics, technicalization, specialization, and intensification. A monitoring system with the same terminal is established to forecast the natural environment of natural disasters and agricultural growth (Xiong et al. 2017). Combined with the research results of the impact of satellite remote sensing image technology on agricultural climate change, we can see that the change of agricultural meteorology will affect crop yield, planting and harvesting time, the growth state of crops, the quality of crops, and even diseases and insect pests have a certain degree of influence, so the study of Agrometeorology is also conducive to the promotion of the development potential of agricultural products, strengthen the use of monitoring system and satellite remote sensing image technology, timely forecast the meteorological situation, and monitor the growth status of crops in real time, which is conducive to the development of agriculture Geerken and Ilaiwi (2004). To prevent the adverse effects and losses caused by meteorological disasters is conducive to improving the quality of agricultural products.

#### Strengthen the construction of agrometeorological service system

The establishment and improvement of automatic and systematic Agrometeorological forecast system is conducive to the development of agricultural characteristics, specialization and intelligence, as well as the development of special and new agricultural industries. Strengthening the forecast and early warning of meteorological disasters and formulating systematic risk prevention measures are conducive to the construction of crop growth environment and the improvement of crop product quality (Zhao et al. 2015). Therefore, it is necessary to strengthen the construction and optimization of agricultural meteorological forecast system. At the same time, it is also necessary to enhance the accuracy of the forecast information in the agrometeorological forecasting system and to connect with the agricultural meteorological monitoring system and big data, so as to improve the linkage ability of early warning and prevention. According to the meteorological changes, we can also formulate a set of agricultural planting scheme that adapts to local conditions, develop characteristic agriculture in different places and different climatic environments, and strengthen the network design the supervision of the equipment, real-time transmission of information.

#### Deepen the integration of different departments and disciplines

It is necessary to strengthen the exchange, communication, and discussion on technology and research with professional departments, scientific research departments, and famous colleges and universities. At the same time, cooperation and exchange between agricultural industry departments such as agriculture, forestry, animal husbandry, and fishery should be strengthened (Luo et al. 2017). At the same time, the influence of agricultural meteorology, natural environment, human activities, plant growth and development, animal breeding, irrigation facilities, fertilizer research, and development and utilization should be combined with agriculture The development of technology will integrate and transform different technologies and knowledge, take the essence to remove the dross, and improve the favorable influence of agrometeorological on the development of agricultural industry.

### Strategies to improve the supply and management capacity of emergency materials for agriculture, rural areas and farmers

In the “three rural” issues, the management of emergency materials is an important issue, and the supply of emergency materials is a crucial issue in the management of emergency materials. To strengthen the supply of emergency supplies, we need to accurately calculate the quantity of purchase through existing data. This practice is the most favorable measure to strengthen management, which can effectively enhance the supply of emergency materials ability.

#### Establishment of emergency material supply guarantee reserve system

There are many essential parts in the agricultural emergency system, one of the most important part is the management of emergency materials. In emergency rescue, the most important thing is to improve its timeliness and effectiveness. In order to deliver the rescue materials to the victims in time, the management of emergency materials is the top priority. Because of the effectiveness of management, we will save a certain cost. At the same time, because of the polygon and instability of the rescue area, we also need to move the big data and network connection equipment to manage the emergency materials and accurately know the quantity and storage location of each material. According to this system, we can accurately select the most convenient and fast material storage point for emergency distribution (Gong 2012). Only by combining material management with information technology, can we ensure the timeliness of disaster relief and deliver the materials to the victims as quickly and safely as possible, and at the same time, we can save the transportation cost as much as possible.

In the emergency rescue, the relevant departments should not only ensure the timeliness of material distribution, so that the disaster victims can get the relief materials as soon as possible but also consider the traffic problems. They should not only quickly distribute but also save the transportation cost. In addition, due to the uncertainty of sudden disasters, there are more demands for emergency materials, and more kinds of materials may be needed. Therefore, it is necessary to ensure that the materials are in reserve and budget. Quality should also consider the cost of storing materials (Zhao et al. 2019). Therefore, in the case of various problems, the state and government departments should strengthen the management of emergency materials and disaster emergency disposal capacity, manage from different angles, find some reputable enterprises to store materials, and, at the same time, communicate with these enterprises in time and calculate the quantity of materials, so as to supply materials and ensure that materials can be timely. To the disaster area (Mann 1945), at the same time, when purchasing materials, the protection materials should be distinguished according to different angles, such as protection angle.

#### Optimize emergency material allocation management system

In the future development process, we should realize the importance of emergency materials management, innovate the management means of emergency materials, improve the allocation efficiency of emergency materials, and help people quickly solve the impact of emergencies. In most cases, we cannot accurately know the specific situation of the emergency and cannot accurately estimate the damage of the disaster, so the number of emergency materials will not have a clear indicator, but we can analyze the characteristics of disasters, summarize the past experience in dealing with disasters, and prepare more emergency materials to ensure the needs of disaster treatment. In the process of practice, people should sum up the shortcomings in the past disaster relief process and constantly improve the management system of emergency materials to ensure that people can deploy emergency materials in a short period of time when disasters occur, so as to ensure people’s safety (Masek et al. 2008).

## Conclusion

Through specific analysis, we can know that the management and reserve of emergency materials is related to the ability and efficiency of people to deal with emergencies. Once the allocation of emergency materials has problems, people’s life safety will be threatened. Therefore, we must establish a scientific management system to improve the management efficiency. In the process of agricultural production, climate change often affects people’s agricultural production activities. In order to improve the modernization level of agricultural development, this paper uses the images collected by remote sensing sensors to analyze the specific problems in agricultural production activities, so as to provide help for people to make full use of agricultural climate resources. Through the analysis of remote sensing images, we can know the distribution of agricultural climate resources in the region and use the collected data to build a mathematical model of meteorological analysis, which has practical significance for the development of agricultural modernization in the future. In addition, this study also analyzed the climatic conditions of agricultural production and explained the influence of meteorological factors on vegetation coverage by means of factor analysis and mathematical analysis. Summarizing the research results of this paper can provide effective help for the development of agricultural modernization and can also help relevant departments to improve the management efficiency of emergency materials for agriculture, rural areas and farmers and improve the quality of agricultural development and people’s ability to cope with disasters.
